# Comprehensive Description of *Fusarium graminearum* Pigments and Related Compounds

**DOI:** 10.3390/foods7100165

**Published:** 2018-10-05

**Authors:** Edgar Cambaza

**Affiliations:** 1 Laboratory of Food Process Engineering, Graduate School of Agriculture, Hokkaido University, Sapporo 060-0808, Hokkaido, Japan; edy@bpe.agr.hokudai.ac.jp; Tel.: +81-80-2876-1106; 2 Department of Biological Sciences, Faculty of Sciences, Eduardo Mondlane University, Av. Julius Nyerere, Maputo nr. 3453, Mozambique

**Keywords:** *Fusarium graminearum*, color, pigments, polyketides, carotenoids

## Abstract

Several studies have explored in depth the biochemistry and genetics of the pigments present in *Fusarium graminearum*, but there is a need to discuss their relationship with the mold’s observable surface color pattern variation throughout its lifecycle. Furthermore, they require basic cataloguing, including a description of their major features known so far. Colors are a viable alternative to size measurement in growth studies. When grown on yeast extract agar (YEA) at 25 °C, *F. graminearum* initially exhibits a whitish mycelium, developing into a yellow-orange mold by the sixth day and then turning into wine-red. The colors are likely due to accumulation of the golden yellow polyketide aurofusarin and the red rubrofusarin, but the carotenoid neurosporaxanthin also possibly plays a major role in the yellow or orange coloration. Torulene might contribute to red tones, but it perhaps ends up being converted into neurosporaxanthin. Culmorin is also present, but it does not contribute to the color, though it was initially isolated in pigment studies. Additionally, there is the 5-deoxybostrycoidin-based melanin, but it mostly occurs in the teleomorph’s perithecium. There is still a need to chemically quantify the pigments throughout the lifecycle, and analyze their relationships and how much each impacts *F. graminearum*’s surface color.

## 1. Introduction

*Fusarium graminearum* (teleomorph: *Gibberella zeae*) is a pathogen of maize, wheat, rice, and barley responsible for the disease known as *Fusarium* head blight (FHB) and mycotoxin contamination [1,2]. FHB destroys the grain starch and protein and was responsible for losses of over $2.7 billion in the United States between 1998 and 2000 [2]. The mold’s most common mycotoxins are nivalenol (NIV) and deoxynivalenol (DON) [3], usually occurring together and frequently associated with gastrointestinal disorders, among other health impairments [4]. However, there are other relevant toxins, such as zearalenone (ZEA) [5], an estrogenic compound capable of causing abortion and other reproductive complications [6,7].

There are very few studies comprehensively describing and relating *F. graminearum* surface colors and its pigments, their properties, and biosynthetic or genetic origin, though some were isolated during the 1930s–1960s [8,9,10,11,12]. There was also considerable chemical analysis of *Fusarium* pigmentation in the late 1970s and early 1980s, but this never tried to relate the compounds with the mold’s observable biological phenomena [13]. Recent sequencing of the *F. graminearum* genome and development gene replacement tools allowed major progress in genetic and biochemical studies [13]. Now, it is known that the red pigmentation of *F. graminearum* is due to the deposition of aurofusarin in the walls [2,14], but it is likely to be the combination of several pigments [15,16].

Pigmentation is part of the mold growth process and it can be used as a tool for growth studies as an alternative to the expansion in size [17]. This approach can help overcome spatial constraints in fungal studies or applications, such as the limited size or particular shape of a Petri dish or bioreactor, or even predict toxin production solely by analyzing the mold surface color. For instance, mutants with absence of the pigment aurofusarin seem to produce an increased amount of ZEA [2], and histone H3 lysine 4 methylation (H3K4me) is important for the transcription of genes for the biosynthesis of both DON and aurofusarin [18]. Thus, there is some connection between the production of major *Fusarium* mycotoxins and pigments.

This review aims to identify the major *F. graminearum* pigments described in the literature and summarize what is known so far about them so that future researchers will be able to more comprehensively relate their dynamics and the mold’s color change.

## 2. *F. graminearum* Colors throughout Its Lifecycle

**Figure 1:**
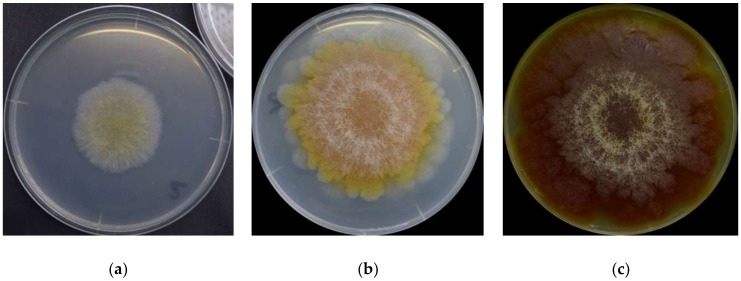
*F. graminearum* surface colors on its (**a**) 3rd day, (**b**) 6th day, and (**c**) 16th day.

It is first important to know that there is no single set of colors to describe *F. graminearum* throughout its lifecycle. The surface colors change depending on several variables, such as strain, maturity, nutrients, temperature, pH, water activity, light exposure, and aeration [8,10,16,17]. Ashley et al. [8] mentioned early studies identifying pH as the main determinant of *Fusarium* colors, “so that one and the same culture may be orange or yellow colored at an acid reaction, the color changing to red or blue when the medium becomes alkaline”. Medentsev et al. [19] said that the biosynthesis of naphtoquinones (major secondary metabolites, including pigments) is the mold’s main response to stress. *F. graminearum* has different types of pigments, all with distinct properties [8,15,20,21,22], from which we have to expect numerous combinations and the resulting chromatic attributes. For instance, the teleomorph was found to have violet pigmentation in its perithecia [23]. Thus, it is impractical to summarize all possibilities. For this reason, this description will simply focus on the mold grown on yeast extract agar (YEA) at 25 °C as an example (Figure 1). The isolate was obtained from the Catalogue of the Japan Collection of Microorganisms (JCM), where it is registered as the teleomorph *Giberella zeae* (Schwabe) Petch, and it was isolated by Sugiura [24] from rice stubble in Hirosaki, Aomori Prefecture, Japan.

YEA is a highly nutritive medium containing agar as a solidifier, peptic digest of animal tissue, and yeast extract, and is thus rich in nitrogenous compounds, vitamin B, and other nutrients [25]. Furthermore, yeast extracts do not seem to affect the quality or level of aurofusarin, a major pigment, by any *Fusarium* species [26]. This is important because it is desirable to use the mold’s original coloration in the studies, without changing it much, because of the nutrients available. 

*F. graminearum* colors change in a very consistent and predictable pattern [17]. At a glance, the mold germinates as a pale mycelium and starts to acquire a yellowish coloration between its third and fourth day. It attains its full orange tone on the sixth day and then shifts to dark wine red by the 16th day. The color distribution is heterogeneous: it forms a radial gradient, with the center more intensely colored and increasingly pale surroundings. Kim et al. [1] described the *F. graminearum* as a “yellow to tan mycelia with the white to carmine red margins”, certainly depending on the condition in which it grows. A dominant red color tends to become more evenly distributed as the fungus ages, and alternated concentric layers of white and red rings close to the center become increasingly more evident. The white rings are hairy and seem to be formed of colorless hyphae. The medium’s color change (Figure 1c) from pale to yellow is due to the accumulation of aurofusarin [14]. 

Recent analysis based on red, green, and blue (RGB) channels taken for *F. graminearum* photographs shows that the three color components are positively correlated and all exhibit a third-degree polynomial trend when measured throughout the first 20 days of its lifecycle, and it makes the colors a potential tool to replace size-based measurements for growth studies and to predict toxin production [17].

## 3. Major *F. graminearum* Pigments

Most of what is known about the pigmentation of *F. graminearum* comes from studies on *F. culmorum*, *F. aquaeductuum*, *F. fujikuroi*, and *F. oxysporum*, and eventual confirmation that the pigments occur across species [15]. Such studies are aimed primarily at enhancing pigment production for the dye industry as an alternative to synthetic counterparts [27]. A pioneer study by Ashley et al. [8] identified aurofusarin, rubrofusarin, culmorin, and their derivatives among the pigments. Since then, others have been mentioned, including perithecial melanin [28] and carotenoids [15]. The most relevant carotenoids from *F. graminearum* are perhaps neurosporaxanthin and torulene [14,15,21].

*F. graminearum* pigmentation is very complex, but most pigments have similar colors, ranging from yellow and orange to red. Thus, it is perhaps difficult to know how much each pigment contributes to its color. Yet, the literature points towards aurofusarin and neurosporaxanthin, and possibly also rubrofusarin, as the ones impacting *F. graminearum*’s surface color the most. No source has simultaneously covered non-carotenoid and carotenoid pigments and the ones describing each of these classes showed the tendency to state the respective compounds as the main source of coloration, maybe because the pigments have similar colors. It would be a good idea to find out which contributes the most to the coloration, perhaps by experimentation based on distinctive properties of the pigments. For instance, carotenoids are expected to be reactive to light, but as far as the literature has shown, polyketides such as aurofusarin and rubrofusarin are not likely to change considerably in the presence and absence of illumination. By simple observation, aurofusarin appears to be predominant because *F. graminearum* specimens grown in dark and illuminated settings do not seem to present different coloration when maintained at the same temperature.

In any case, the color change has previously been demonstrated to follow a predictable trend, disregarding the pigments involved. Thus, whichever the dominant pigments, they follow a consistent pattern over time. It is still difficult to advocate if the changes are mostly due to variations in the proportion of different pigments or chemical reactions leading to changes of the same compound into its derivatives, just like the case of aurofusarin at different pH settings. It could even be simply the breakdown of aurofusarin into rubrofusarin molecules.

There are two more aspects to consider before listing *F. graminearum* pigments or related compounds. Culmorin is colorless, but it is included in this review because it was isolated together for the first time during studies of *Fusarium* pigmentation. Bikaverin and fusorubin are *Fusarium* pigments [19,29,30], but they are not included in the following list because there is very little evidence about their occurrence and impact on the coloration of *F. graminearum*.

### 3.1. Aurofusarin

**Figure 2:**
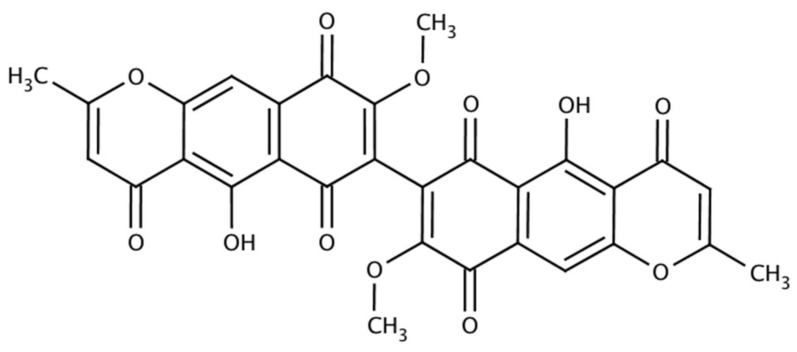
Structure of aurofusarin. Source: Glentham Life Sciences [33].

Aurofusarin was obtained chemically and isolated from *F. culmorum* before Baker et al. [10] extracted and purified it from a strain of *F. graminearum* Schwabe. It is a dimeric metabolite belonging to the naphthoquinone group of polyketides [13,31], described as a golden yellow-orange or red micro-crystalline pigment in the form of a prism [8,13,20,32], with C_30_H_18_O_12_ as its molecular formula (Figure 2), 570.5 g/mol as its molecular weight, and a melting point over 360 °C [8,33]. It is now assigned as 13191-64-5 under the Chemical Abstracts Service (CAS) [33]. Aurofusarin is the only *F. graminearum* pigment produced under a deficiency of nitrogen, phosphorus, oxidative stress, and the inhibition of respiration [19]. 

Organic solvents can solve aurofusarin moderately, and it becomes yellow in acid and reddish in alkalis [12]. This is a plausible explanation for the color changes throughout *F. graminearum*’s lifecycle (Figure 1); perhaps the mold turns the medium increasingly alkaline and it causes aurofusarin to change its color to red. However, pH ≤ 4 seems to prevent *F. graminearum* from producing aurofusarin [19]. Indeed, pH is the most important regulator of aurofusarin production because most naphtoquinones tend to be cytostatic at a neutral pH level [14]. Yet, temperature and water activity (a_w_) also have an impact on aurofusarin biosynthesis, as they were found to be directly proportional [3].

The species *F. acuminatum*, *F. avenaceum*, *F. crookwellens*, *F. culmorum*, *F. graminearum*, *F. poae*, *F. pseudograminearum*, *F. sambucinum*, *F. sporotrichioides*, and *F. tricinctum* all produce aurofusarin [13]. Different strains of *F. graminearum* produce different quantities under similar conditions [34]. Besides *Fusarium* species, *aurofusarin* can also be isolated from *Hypomyces rosellus* and *Dactylium dendroides* [35]. It is negatively correlated with vegetative growth [1], thus the pigment is expected to be more abundant in differentiated structures. *Aurofusarin* certainly increases the organism’s competitive saprophytic ability due to its antibiotic properties, but it does not help the fungus colonizing the host crops and does not protect the fungus against radiation [2]. The pigment can be extracted using benzene-acetone (4:1) and purified by chromatography on silica gel impregnating with oxalic acid [12,20].

**Figure 3:**
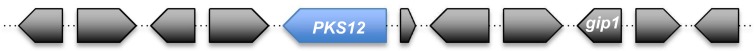
Biosynthetic gene cluster for aurofusarin in *F. graminearum*. Based on Hoffmeister and Keller [39].

The aurofusarin biosynthetic pathway involves several genes in clusters and at least five enzymatic steps, with rubrofusarin as an intermediate [36]. For instance, its synthesis requires the intervention of polyketide synthase genes [37] in a 30 kb cluster (Figure 3), including *PKS12*, *AurR1*, *aurJ*, *aurF*, *gip1*, and *gip2* [36,38] within at least 11 reading frames (FG02320.1–FG02330.1) plus a facilitator transporter gene (FG02331.1) [39]. Among the genes, *AurR1* and *gip2* are believed to be the cluster’s transcription factors [1,13], and the putative laccase *gip1* has been described as potentially responsible for the dimerization of rubrofusarin into aurofusarin [36,40]. More recently, two more genes (*gip3* and *gip8*) were also shown to be important for the dimerization [38]. Frandsen et al. [36] mentioned two more orphan genes (*aurZ* and *aurS*), and *AurT*, an aurofusarin pump, responsible for transporting aurofusarin and rubrofusarin across the plasma membrane.

Though aurofusarin was described before 1937 [8], it has only been regarded as a food and feed contaminant since start of the new millennium [41]. There is considerable literature about the issue [31,41]. It is frequently found in several commodities throughout virtually all climatic regions in the world, sometimes at concentrations as high as 2046 µg/kg to 10,200 µg/kg [31]. Beccari et al. [34] detected 10,400–140,000 µg/kg in Italian samples of durum wheat, Ezekiel et al. [42] mentioned concentrations above 800 µg/kg in chicken feed, and Nichea et al. [43] detected median concentrations of 71.4 µg/kg (2011) and 80.7 µg/kg (2014) in native grass of Argentina, intended for grazing cattle. The latter was found to co-occur with zearalenone.

Aurofusarin is bioactive and it is considered a neglected mycotoxin [31,44]. According to Medentsev et al. [32], it inhibits the growth of some molds and yeasts, and Tola et al. [45] also found it to impair the growth of Red Tilapia (*Oreochromis niloticus* × *O. mossambicus*). Moreover, it was found to be cytotoxic for colon adenocarcinoma cell line HT29 and the non-tumorigenic colon cells HCEC-1CT when at concentrations above 1 µM [31]. It has also shown toxicity for differentiated intestinal porcine epithelial cells (IPEC-J2) when combined with DON [46]. Dvorska, Surai and their colleagues [41,47,48,49] performed a series of studies demonstrating the detrimental effects of aurofusarin on Japanese quail eggs. They found aurofusarin to cause a significant decline of vitamins E and A, total carotenoid, lutein and zeaxanthin, and stimulated lipid peroxidation in the egg yolk. There are many details yet to be clarified regarding the way that aurofusarin affects these compounds. Regarding fats, the authors stated that aurofusarin exhibited an association with a “decrease in the docosahexaenoic acid proportion in the phospholipid, cholesteryl ester and free fatty acid fractions of the egg yolk” and a simultaneous increase in “the proportion of linoleic acid in the phospholipid, free fatty acid and triacylglycerol fractions”. Dvorska [41] also said that aurofusarin reduces the quality of chicken meat, though the mechanism of how it happens is presently unclear.

Regarding the relationship between pigmentation and mycotoxin contamination, there is solid yet scarce literature relating the genetic and biosynthetic origins of aurofusarin and both DON and ZEA [2,18]. As mentioned earlier, Malz et al. [2] demonstrated that mutants for the gene PKS12, unable to produce aurofusarin, produced an increased quantity of ZEA. It suggests that some inhibitory factor for ZEA is related to aurofusarin synthesis, but the mechanism is still to be identified. Furthermore, there seems to be some quantitative relationship between the three compounds, as the combined results of two studies suggest some correlation between them [3,34], though this possibility still requires further investigation. These studies are still in the preliminary stages, but they demonstrate that some genetic factors are common for aurofusarin and these mycotoxins. If such factors have a similar influence on aurofusarin and the toxins, variations in the quantity of aurofusarins might theoretically be used to predict the quantity of DON or ZEA. Thus, there is a possibility that *F. graminearum*’s surface color can be a good predictor of toxicity. Furthermore, aurofusarin is a toxin itself. It can surely be quantifiable through the mold’s surface color.

### 3.2. Rubrofusarin

**Figure 4:**
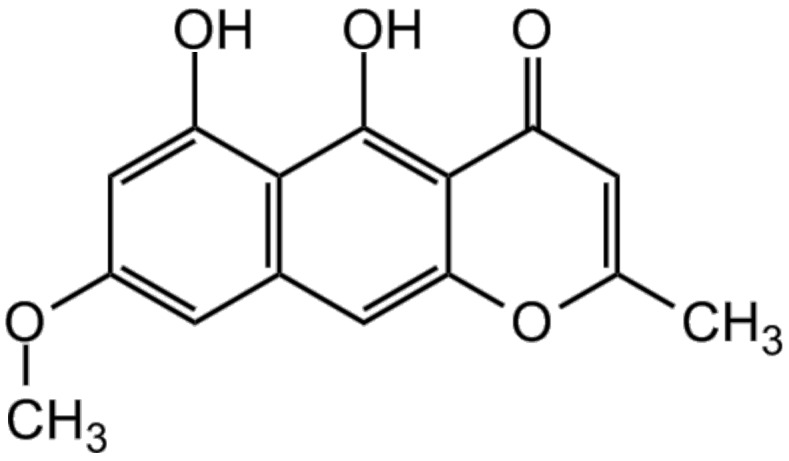
Structure of rubrofusarin. Source: BioViotica [50].

Rubrofusarin (CAS: 3567-00-8) is a crystalline polyketide red-orange pigment [8,20] usually found in the form of needles [10,50]. Demicheli et al. [51] described it as a powder. Rubrofusarin belongs to the class of naphthopyrones and resembles the aurofusarin monomer [13], consisting of a mono methyl ether [8] (Figure 4). Indeed, the biosynthesis of both pigments seems connected because different levels of aeration produce distinct proportions of rubrofusarin and aurofusarin [22]. Thus, the logic behind aurofusarin as a predictor of *F. graminearum* toxicity mentioned above in theory is likely to be applicable to rubrofusarin.

Stout et al. [9] and Tanaka et al. [52] were the first investigators presenting its structure in 1962. According to them, rubrofusarin’s molecular formula is C_15_H_12_O_5_ and the melting point is 210–211 °C. The molecular weight is 272.3 g/mol [50]. Rubrofusarin is insoluble in water, but it is soluble in ethanol and dimethyl sulfoxide (DMSO) [51]. Its color does not respond to pH, unlike aurofusarin [8]. Furthermore, it has chelating properties, forming complexes with Mg^2+^, Al^3+^, Fe^3+^, Ni^2+^, and Cu^2+^ in a solid state and aqueous medium [53,54,55].

Several studies mention rubrofusarin or derivatives isolated from different sources, usually fungi [56], or plant roots or seed [55], for pharmacological purposes. Ashley et al. [8] published a pioneer report on the isolation of rubrofusarin from *F. culmorum* and *F. graminearum*. From the fungus *Guanomyces polytrix*, Mata et al. [57] isolated rubrofusarin B, a variation of the compound in which methyl ether replaces the hydroxy group at position 6 [58]. Another rubrofusarin producing mold is *Aspegillus niger*, also known for producing ochratoxins and consequently causing the Balkan nephropathy [59]. Regarding plants, Rangaswami [60] isolated the compound from *Senna tora* in India, and Oliveira et al. [61] obtained the rubrofusarin glycoside from the softwood of *S. macranthera* in Brazil. *Senna* comprises a diverse genus of native leguminous throughout the tropics [51]. *Berchemia polyphylla* var. leioclada, a woody deciduous plant abundant in China, produces at least three rubrofusarin glycosides [62]. Other rubrofusarin producing species are *Paepalanthus bromelioides* [63] and *Flavoparmelia euplecta* [64]. Moreover, it is reasonable to think that virtually all aurofusarin producing organisms also have the potential to produce rubrofusarin because the latter pigment is an intermediate of the aurofusarin biosynthetic pathway [36].

*F. graminearum* synthesizes rubrofusarin through a polyketide chain intermediate by condensing seven acetate units [65]. Deletion mutants of *AurR1* and *PKS12* cannot synthesize rubrofusarin, exactly as it happens to aurofusarin [13]. Furthermore, the FG12040 protein is also responsible for rubrofusarin synthesis, and the process is inhibited in mutants without the gene *cch1*, known as a calcium ion channel encoder [66]. Rugbjerg et al. [67] confirmed the intervention of the genes mentioned and the metabolic pathway by reconstructing it using *Saccharomyces cerevisiae*. They “paved the way” for industrial production of the pigment.

There is plenty of pharmaceutical potential of rubrofusarin and derivatives to be unlocked, and some of it has already been demonstrated. Rubrofusarin has antimycobacterial, antiallergic, and phytotoxic properties to herbs *Amaranthus hypochondriacus* and *Echinochloa crus-galli* [1,38,40,59]. According to Alqahtani et al. [68], rubrofusarin can enhance the cytotoxicity of paclitaxel (PTX) against the adriamycin-resistant breast cancer cell line MCF-7adr. Still regarding cancer research, rubrofusarin B was cytotoxic for the colon cancer cell line SW1116 [59], and rubrofusarin presented complete inhibitory ability against human DNA topoisomerase II-α, meaning that it can be used to develop potential anticancer and antiviral drugs [56]. Jing et al. [62] found rubrofusarin glycosides to have antioxidant properties, one of which was even stronger than vitamin C. The pigment was also found to exhibit estrogenic activity [69]. Rubrofusarin bioactivity has led Moreira et al. [55] to effectively demonstrate its fitness to be applied as a fluorescent probe.

### 3.3. Culmorin

**Figure 5:**
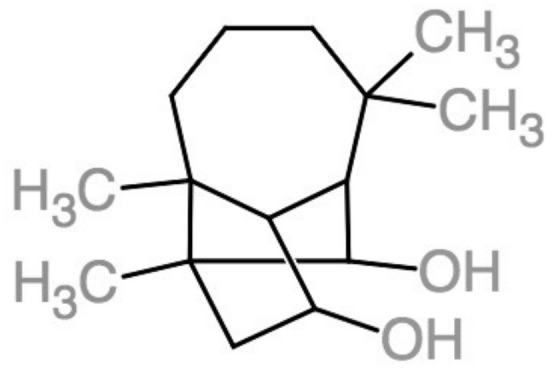
Structure of culmorin. Made on ChemDoodle [77], based on Nara Institute of Science and Technology [78].

Culmorin (CAS: 18374-83-9) is a natural colorless metabolite found in various *Fusarium* species [8,20,70,71], with C_15_H_26_O_2_ as its molecular structure (Figure 5), first isolated by Ashley et al. [8] and later established by Barton et al. [11]. Culmorin is defined as a longifolene sesquiterpene diol with a tricyclo-[6.3.0.0] undecane skeleton [72], and its molecular weight is 238.3 g/mol [73]. It seems to be biosynthesized from *trans*-farnesyl pyrophosphate [70] and compounds closely related include hydroxyculmorins, culmorone, and hydroxyculmorone [74,75,76]. Technically, it is not a pigment in the sense that it has no color, as already mentioned, but it was initially isolated during pigment studies together with aurofusarin and rubrofusarin [12], possibly because they share some chemical properties.

So far, the culmorin producing *Fusarium* species mentioned in the literature include *F. graminearum*, *F. culmorum*, *F. crookwellense* (*F. cerealis*), *F. venenatum* [79], and more recently, *F. praegraminearum*, a basal species of the *F. graminearum* complex [80]. Laraba et al. stated that 77% of *F. culmorum* genotypes were capable of producing culmorin in Algeria, and most were around the subtropical areas. Besides *Fusarium*, the marine fungi *Leptosphaeria oreamaris* [81] and *Kallichroma tethys* also synthesize culmorin [79]. 

The biosynthesis of culmorin requires the gene *CLM1* (GenBank: GU123140.1), responsible for encoding a longiborneol synthase for the compound’s pathway [82,83]. The gene *CLM2* encodes the cytochrome P450 and this is responsible for the subsequent hydroxylation of longiborneol. Culmorin was also synthesized in vitro using tetrahydroeucarvone [81]. Citric and lactic acids (5%) seem to attenuate the synthesis in feed, and this also happens to other *Fusarium* metabolites, including DON [84].

*F. graminearum* grown in durum wheat was found to produce culmorin at concentrations exceedingly high (2.5–14 g/kg) in central Italy, and there were considerable variations between strains [34]. It was also detected in Norway at median concentrations of 100 µg/kg (wheat), 292 µg/kg (barley), and 2000 µg/kg (oats) [85]. Similar results were found in an ensemble study from Austria, Denmark, and Hungary [44]. In Cameroon, Abia et al. [86] detected culmorin in cereals, nuts, and derivatives at a median concentration of 100 µg/kg. Generotti et al. [87] demonstrated that culmorin from contaminated wheat flour can endure an entire biscuit baking process, with final levels up to 92 µg/kg in baked biscuits, corresponding to a percentage of heat degradation between 25% and 80% at 180 °C. Their final products also exhibited 15-hydroxy-culmorin. Culmorin and derivatives also seem to resist the brewing process, even after the treatment of the substrates with the fungicide Prosaro^®^ 250 [88].

Culmorin has mild [79] antifungal activity against several molds, particularly wheat and corn parasites [81]. It is also phytotoxic [72]. Regarding the fungal-plant interaction, culmorin presented a correlation with the amount of lutein in durum wheat contaminated with *Fusarium* [89], and this could be a way to empirically estimate the extent of contamination. Furthermore, it is frequently detected with DON [70,76,87], usually in a quantity three-fold higher, though some variables can influence this ratio [72]. However, since culmorin is colorless, theoretically, it cannot be directly measured through *F. graminearum* surface color. Culmorin and other *F. graminearum* secondary metabolites seem to enhance DON toxicity in caterpillars [90] and pigs [76,91]. Yet, culmorin itself is weakly toxic and it is negative to the Ames test of mutagenicity [79].

### 3.4. Black Perithecial Pigment

**Figure 6:**
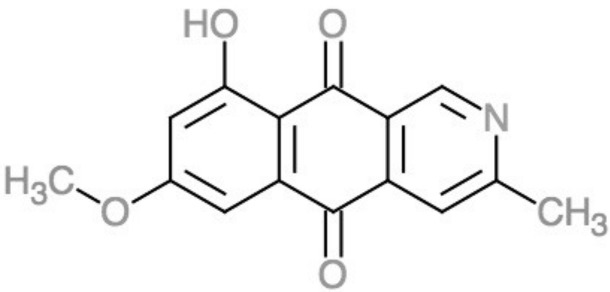
Structure of 5-deoxybostrycoidin. Made on ChemDoodle [77], based on Frandsen et al. [28].

*Fusarium* is among the deuteromycota now known for having a teleomorphic ascomycota called *Gibberella*, and as such it develops a reproductive fruiting body called perithecium. The black perithecial pigment, sometimes described as dark blue, violet, or purple [28,92], is almost restricted to the fruiting body [93], though the producing gene is even present in *Fusarium* species with an unknown teleomorph [23]. The pigment’s nature is still under study and only recently has been related to fusorubins [94] and described as ″5-deoxybostrycoidin-based melanin″ [28], with C_15_H_11_NO_4_ as the molecular formula [95] (Figure 6).

Several *Fusarium* species, including *F. graminearum*, *F. verticillioides*, and *F. fujikuroi* [28,94], produce the blackish perithecial pigments, and appear to be related to an ancestral highly conserved gene cluster [96]. The fungus *Nectria haematococca* also showed the ability to produce 5-deoxybostrycoidin [95]. In *F. graminearum*, the six-gene *PGL* cluster, particularly the gene *PGL1* or *PKS3*, seems to be associated with the production of the polyketide synthase responsible for biosynthesis of the black perithecial pigment [37,97]. *PGL1* is related to a transcription-associated protein (TAP) cluster called TC3 [93]. Studt et al. [94] demonstrated an association between the dark pigments and fusarubin under the intervention of the so-called *fsr* gene cluster.

Evidence suggests that this pigment is important for UV or desiccation protection during the differentiation of perithecia and ascospores [37], and this role is a function of its chemical composition, free radical quenching, and the distribution of the pigment through the perithecial surface [98].

### 3.5. Carotenoids

Natural carotenoids comprise a family of more than 750 natural lipophilic terpenoids, several of which are produced by fungi, though they are not essential for these organisms [15]. Carotenoids are common in molds and they contribute to the yellow, orange, and reddish coloration [99]. They have been produced industrially and are widely used as food and feed additives [100]. 

An important characteristic of carotenoids worth mentioning is their sensitivity to light. As Avalos et al. [15] stated, illumination induces the synthesis of carotenoids through the transcriptional induction of structural genes in *Fusarium*. Jin et al. [21] identified neurosporaxanthin and torulene as the most relevant, but other carotenoids that likely play a minor role in *F. graminearum*’s color pattern are torularhodin, β-carotene, γ-carotene, ζ-carotene, and β-zeacarotene [101,102,103].

#### 3.5.1. Torulene

**Figure 7:**
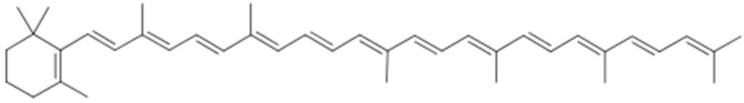
Structure of torulene. Source: Royal Society of Chemistry [106].

Torulene (CAS: 547-23-9) is a natural carotenoid of importance for industrial application [104,105], with C_40_H_54_ as its molecular formula and 534.9 g/mol as its molecular weight [105]. As Figure 7 shows, it has 13 double bonds, a β-ionone, and a long polyene chain [104]. In petroleum ether solution, torulene has a pinkish-red color, depending on the concentration [103]. 

*Neurospora crassa* is the most well-known producer of torulene, but it is also synthesized by *F. fujikuroi* [107]; *F. graminearum* [21]; the red yeasts *Sporidiobolus pararoseus* and *Rhodotorula glutinis* [108] and related organisms; and the genera *Cystofilobasidium*, *Dioszegia*, *Rhodosporium*, and *Sporobolomyces* [103]. These organisms probably produce it as protection against photo-oxidation and free radicals [109].

Geranyl-geranyl diphosphate is the precursor of torulene, and it is transformed under the intervention of two enzymes called AL-2 and AL-1, produced by eponymous genes [107]. The biosynthesis is mostly influenced by nutrients, especially the sources of carbon and nitrogen, but it also responds to other factors such as aeration, temperature, acidity, exposure to radiation, and the presence of chemicals such as alcohols [103].

There is very little information on torulene’s bioactivity and nutritional value, perhaps because it is rare in food [110], but its nature, structure, and sparse evidence provide some hints. Animal studies demonstrated its safety to be used as a food additive [104], and Kot et al. [103] added that it can also be used as feedstock and a cosmetic additive. The presence of a non-substituted β-ionone ring makes torulene pro-vitamin A, which is likely to present higher antioxidant or free radical scavenging activity than β-carotene, higher reactivity in aqueous solutions, more efficient electron transfer-reactions than lycopene [108], and there is evidence of anti-prostate cancer activity [104].

#### 3.5.2. Neurosporaxanthin

**Figure 8:**
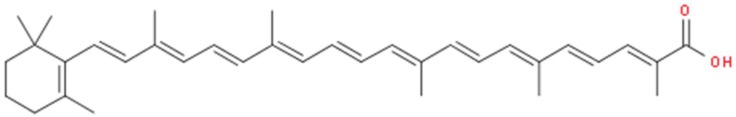
Structure of neurosporaxanthin. Source: Royal Society of Chemistry [113].

Neurosporaxanthin (CAS: 2468-88-4), or β-apo-4′-carotenoic acid, is carboxilic apocarotenoid xanthophyll [99,111], with C_35_H_46_O_2_ as its molecular formula (Figure 8) and 498.8 g/mol as its molecular weight [107,112]. Neurosporaxanthin carries the name of *Neurospora crassa*, from where it was originally isolated, but *Fusarium, Verticillium*, and *Podospora* species also synthesize it [99,111].

Prado-Cabrero et al. [114] described neurosporaxanthin as a “cleavage product of torulene”. The biosynthesis initiates from the condensation of two geranylgeranyl pyrophosphates into phytoene and following desaturations [99,100]. The cleavage of torulene into neurosporaxanthin requires the carotenoid oxygenase CAO-2, first resulting in β-apo-4’-carotenal, the aldehyde of neurosporaxanthin [107]. The major genes involved in the process are *carRA*, *carB*, *carT*, and *carD* [99]. The latter gene is in a different cluster.

## 4. Conclusion

In summary, it is reasonable to assume the possibility of using *F. graminearum*’s surface color to estimate how much toxin the mold is producing and to possibly estimate its potential bioactivity, but there is still a lot to be investigated in relation to *F. graminearum* and several other molds. It is necessary to catalog the pigments produced by each species and clarify their biosynthetic relationships to prevent information “gaps”. For instance, a trichothecene-producing mold is perhaps likely to also produce culmorin, and there are also parallelisms between aurofusarin and rubrofusarin or torulene and neurosporaxanthin. It is true that commercial demand is frequently a major driving force for research and sometimes it is not merely choice or curiosity leading investigators, but it is important to always try to build a very cohesive body of knowledge from which other researchers can craft their own contributions.
